# The Prevalence of Vitamin D Deficiency in Adults in Kermanshah, Western Iran

**Published:** 2018-02

**Authors:** Mazaher RAMEZANI, Masoud SADEGHI

**Affiliations:** 1. Molecular Pathology Research Center, Emam Reza Hospital, Kermanshah University of Medical Sciences, Kermanshah, Iran; 2. Medical Biology Research Center, Kermanshah University of Medical Sciences, Kermanshah, Iran; 3. Students Research Committee, Kermanshah University of Medical Sciences, Kermanshah, Iran

## Dear Editor-in-Chief

Vitamin D deficiency (VDD) is a major public health problem around the world (1). High prevalence of VDD is present in Iranians of different age groups (2). VDD is considered as a 25-hydroxyvitamin D level ≤20 ng/mL (50 nmol/l) in studies (3) and 25(OH) vitamin D levels>30 ng/ml have been proposed as “vitamin D sufficiency” (4, 5). The major cause of VDD is the lack of a full understanding that sun exposure in moderation is the major source of vitamin D of most humans. Very few foods naturally contain vitamin D and foods that have vitamin D, are often insufficient to satisfy either a child's or an adult's vitamin D requirement (6). VDD can culminate in bone loss from decreased vitamin D–mediated intestinal calcium (Ca) absorption (7). Patients with severe and long term VDD may show overt hypocalcemia and/or hypophosphatemia, but this is rare (7).

During Oct 2015 to Jun 2016, 2102 adult individuals (≥20 yr) referred to the Laboratory of Mahdieh, Kermanshah, western Iran. Age, sex and vitamin D level were checked in all patients, but Ca and phosphorus (P) levels checked for 739 individuals. We used ELISA method for measurement of 25(OH) vitamin D in serum. The vitamin D level was defined to three different degrees [sufficiency (>30 ng/mL or 75 nmol/L), insufficiency or mild deficiency (20–30 ng/mL or 50–75 nmol/L) and deficiency (<20 ng/mL or <50 nmol/L)] (4, 8). Moreover, we used Photometric assay for measurement of Ca level in serum and Phosphomolybdate, UV, Endpoint method for measurement of P level.

Data were analyzed with IBM SPSS version 19 software (SPSS Inc., Chicago, USA). P<0.05 was considered statistically significant.

The mean age was 45.3 yr (range, 20–93 yr) that 88.3% had age<65 yr and 17.9% were men. Out of 2102 adult individuals, 739 individuals were checked for Ca and P levels. The mean serum vitamin D, Ca and P levels (ranges) were 27.4 ng/mL (0.4–188 ng/mL), 10.00 mg/dL(7.80–10.89 mg/dL) and 3.60 mg/dL(2.10–5.10 mg/dL), respectively. There was not a significant difference between the variables in two genders (*P*>0.05). Maximum of overall deficiency rate (%) was in 26–29 yr, 40–49 yr in females and 30–39 yr in males. Minimum of overall deficiency rate (%) was in 60–69 yr that this range was similar in females and also in males.

**Fig. 1:**
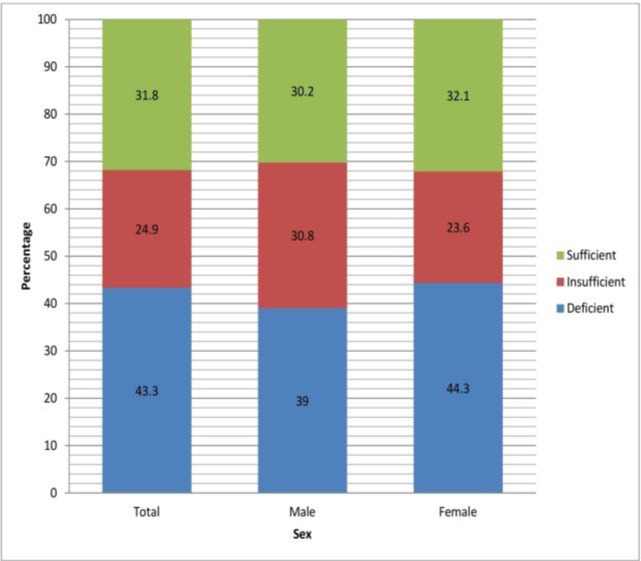
The prevalence rate (%) of different degrees of vitamin D (ng/mL)

The overall prevalence rate of VDD (both genders) was 43.3% (Fig. 1) that for males was 39% and 44.3% for females (*P*=0.013). The overall prevalence rate of VDD and insufficiency (inadequate vitamin D) was 68.2%, including 69.8% for men and 67.9% for women (*P*>0.05). Therefore, the VDD was more in females compared with males.

There were significant differences between age and P level with different degrees of vitamin D (*P*<0.001 and *P*=0.043, respectively) (Table 1). There were no significant correlations between P and Ca levels with vitamin D level.

**Table 1:** The correlation between variables and different degrees of vitamin D (n=2102)

***Variables***	***Deficient***	***Insufficient***	***Sufficient***	P-value^*^
Age (yr)	41.2±13.6	44.9±14.0	51.3±15.2	<0.001
Ca (mg/dL), n=739	10.02±0.48	9.96±0.50	10.1±0.48	0.100
P (mg/dL), n=739	3.52±0.39	3.61±0.41	3.56±0.39	0.043

* One-way ANOVA

The results showed that nutrition experts, health professionals and doctors should note that even in sunny areas should encourage healthy people to consume the enriched foods and guide patients to use oral supplements of vitamin D and also training of people for wearing the suitable clothes and time going out based on seasons.
